# Cell Phenotypes in Human Amniotic Fluid

**Published:** 2009-07

**Authors:** D.A. Davydova, E.A. Vorotelyak, Yu.A. Smirnova, R.D. Zinovieva, Yu.A. Romanov, N.V. Kabaeva, V.V. Terskikh, A.V. Vasiliev

**Affiliations:** 1 Koltzov Institute of Developmental Biology, Russian Academy of Sciences;; 2 Russian Cardiology Research-and-Production Complex

## Abstract

Stem cells capable of long-term proliferation and differentiation into different cell types may be a promising source of cells for regenerative medicine. Recently, much attention has been paid to fetal stem cells, among which are cells from amniotic fluid (AF). We have isolated amniotic stem cells from 3 AF samples. Flow cytometry, RT -PCR and immunohistochemistry have shown that these cells express mesenchymal (CD90, CD73, CD105, CD13, CD29, CD44, and CD146), neural (≤3-tubulin, Nestin, and Pax6), epithelial (keratin 19 and p63) markers and also markers of pluripotency (Oct4, Nanog, and Rex-1). Transplantation of the cells to nude mice does not lead to tumor formation. Thus, putative stem/progenitor cells from AF are capable of long-term proliferation in vitro and the profile of gene expression led us to speculate that they have greater differentiation potential than mesenchymal stem cells and may be useful for cell therapy.

## INTRODUCTION

AF has been used in prenatal diagnosis of genetic diseases for more than 70 years [1]. It contains a heterogeneous population of cells, which includes cells from fetal skin, respiratory, digestive, and urinary tracts, as well as cells from the amniotic membrane. Most of these cells are differentiated and have a low proliferative potential [17, 21]. Recent data seem to indicate that AF contains cells which can proliferate for extended periods of time and can differentiate in vitro into different cell types. Based on the fact that these cells express such markers as CD73, CD90, CD105, CD44, and CD29, several researchers consider them as MSCs [20; 16]. Interestingly, cells isolated from AF express neural markers, such as Nestin, ≤3-tubulin, GFAP, NEFH, as well as several markers of ESCs, such as SSEA-4, Oct4, and Nanog [13; 17; 21]. These cells exhibit osteogenic, adipogenic, myogenic and neural differentiation; they can also differentiate into hepatocytes and endothelial cells [20; 7; 21; 6; 12; 25; 26]. Thus, the available data suggest, on the one hand, that cells from AF are intermediate in their differentiation potential (between embryonic and adult stem cells) and, on the other hand, the possibility that AF culture contains several distinct cell types (i.e. population heterogeneity). In order to assess this possibility, a further detailed investigation of the population structure is needed, which implies extensive data on the gene expression profile. 

Obtaining AF is a very simple and safe procedure; the cells from AF are relatively easy to isolate and cultivate, and they show little immunogenicity and higher proliferative potential than that of adult stem cells. Also, AF cells can differentiate into the derivatives of the three germ layers and do not form teratomas after transplantation. All these facts suggest that AF can be an alternative source of stem cells for cell therapy [14; 7; 19]. Also, the possibility of obtaining cells which express several pluripotency markers evade the ethical concerns arising in human ESCs research. The goal of this study was to investigate the proliferative potential of cells isolated from AF and to analyze the expression of certain tissue-specific genes and stem cell markers. 

## MATERIALS AND METHODS

### AF CELL CULTURE

Samples of AF (10 ml) were obtained from three donors via amniocentesis performed at 16-20 weeks of pregnancy in Snegirev Obstetrics and Gynaecology Clinic, Moscow. The cells were collected by centrifugation (10 min, 1100 rpm) and cultured in ≤-MEM medium (Gibco, United States) supplemented with 15% ES-FBS (HyClone, United States), 1% glutamine (Invitrogen, United States), 18% Chang B and 2% Chang C (Irvine Scientific, United States), and 1% penicillin/streptomycin (Sigma, United States) at 37°C with 5% humidified CO2. Cells were replated at 1:3 every 2nd or 3rd day, when they grew to confluence. 

### Flow Cytometry

Expression of the surface antigens in AF cells (passage 7) was assessed using a flow cytometer (Becton Dickinson FACSCalibur, United States). The cells were trypsinzed and stained with fluorescein isothiocyanate- (FITC ) or phycoerythrin- (PE) conjugated antibodies against CD13, CD29, CD44, CD106, CD73, CD54, CD45, CD34, CD146, CD90, CD105, CD71, HLA-A,B,C, and HLA-DR,DP,DQ (BD Pharmingen, United States). FITC - or PE-conjugated immunoglobulins of the same isotype were used as controls. Mouse antibodies against keratin 19 (Millipore, United States) with secondary Alexa Fluor 488 (Molecular Probes, United States) antibodies were used to assay keratin expression. Staining without primary antibodies and isotypic controls were also performed. 

### RT-PCR

Total RNA extraction was performed with TR I® Reagent (Sigma, United States) in accordance with the manufacturer's protocol. mRNA was isolated by using magnetic beads (Sileks, Russia). The first cDNA strand was synthesized with the M-MLV reverse transcriptase (Sileks, Russia). cDNA libraries were normalized to the housekeeping gene RPL19. The PCR primers were constructed with DNAStar software and located in different exons. Information on the structure of the studied genes was obtained from the National Center for Biotechnology Information (NCBI, GeneBank, United States). Primer sequences are listed in Table 1. PCR with specific primers was performed with ColoredTaq-polymerase (Sileks, Russia) on a Mastercycler (Eppendorf, Germany). PCR fragments were separated by electrophoresis in 1% agarose gels and analyzed on a UV gel analyzer (®BIO RAD, United States).

**Table 1:** Primers

Gene	Primer sequence
Rpl19	5' agggtacagccaatgcccga 3' 5' ccttggataaagtcttgatgatc 3'
CD90	5'acctggccatcagcatcgct3' 5'gaaatccgtggcctggagga 3'
β_3_-tubulin	5'cagtgcggcaaccagatcgg 3' 5'caggtcagcgttgagctggc 3'
Nestin	5' aggaggatgtaccaccagtgc 3' 5' caccaatgatgtctgcccct 3'
Nucleostemin	5'gcatgacctgccataagcgg3' 5'ctgtccactctggacaatggc3'
Pax6	5' gtcatcaataaacagagttcttc 3' 5' cgattagaaaaccatacctgtat 3'
Keratin 19	5'gatcgaaggcctgaaggaag3' 5'atgctcagctgtgactgcag3'
p63	5'gagccgtgaattcaacgagg3' 5'tccgaaacttgctgctttctg3'
CD117	5'gtgggcgacgagattaggctg3 5'cgcgtttcacacttttgatcatg3'
Oct4	5'cgaccatctgccgctttgag3' 5'ccccctgtcccccattccta3'
Nanog	5'gtgtggatccagcttgtccc 3' 5'ctgcgtcacaccattgctattc 3'
Rex1	5'gctggagcctgtgtgaacag3' 5'atcacataaggcccacaccg3'
Stella	5'gcctagtgttgtgtcaagac3' 5'ggtgcaagaataagatttatggc3'
Sox2	5'acagcccggaccgcgtcaag 3' 5'tctgcgagctggtcatggag 3'

### Immunohistochemistry

Cells for immunohistochemistry were taken at the 11th passage, fixed with 4% paraformaldehyde and incubated overnight at + 4°C with antibodies against CD34 (mouse, 1:200, Millipore, United States), CD105 (mouse, 1 : 50, Millipore, United States), CD49d (mouse, 1 : 50, Millipore, United States), STR O-1 (rabbit, 1 : 100, R_D Systems, United States), keratin 14 (mouse, 1:20, Novocastra, Germany), keratin 19 (mouse, 1 : 50, Millipore, United States), p63 (mouse, 1 : 50, BD Pharmingen, United States), ≤3-tubulin (mouse, 1:300, Millipore, United States), NF (Neurofilament) (mouse, 1:10, ICN , United States), Pax6 (mouse, 1:100, Millipore, United States). After that, the cells were washed with PBS and incubated with secondary Alexa Fluor 488 or Alexa Fluor 546 antibodies (Molecular Probes, United States) at room temperature for 1 h. Cell nuclei were stained with DAPI (VECTASHIELD mounting medium for fluorescence with DAPI, Vector Laboratories, United States). 

### Immunodeficient Animal Transplantation

In order to study the ability of AF cells to form teratomas, immunodeficient Nude mice were used (Pushino animal nursery). The animals were subcutaneously injected with 3° x 106 5th passage cells suspended in 100 microliters of serum-free ≤-MEM medium (suspension concentration 30 ° x 106 cells/ml). Control animals were injected with 100 microliters of suspension of 2nd passage stem cells from human adipose tissue (45 ° x 106 cells/ml) (negative control) and 100 microliters of 65th passage mouse ESCs suspension (20 ° x 106 cells/ml) (positive control). Each group consisted of three animals. Animals were withdrew from the experiment when tumors were detected (positive control) or 11 weeks after injection (experimental group and negative control). Tumors and animal organs (liver, kidney, spleen, heart, lung, and testicle) were analyzed histologically. 

## RESULTS AND DISCUSSION

### AF Cell Culture 

Three samples of AF from three donors were used in this study. Cultivation was performed in 24-well plates in Chang medium, following the previously described protocol [6] with modifications.

**Fig. 1.**
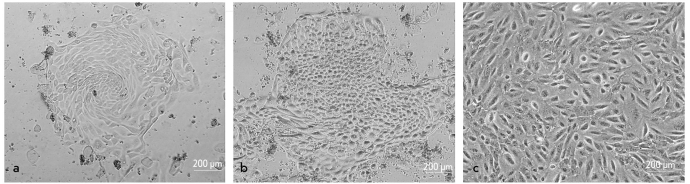
AF cells in culture. (a) Primary fibroblastic cell colony on day 9 of culture. (b) Primary epithelioid cell colony on day 9 of culture. (c) Cells grew to confluence in the 8th-passage culture. (a, b, c) Light field

Centrifugation of AF yielded a heterogeneous population of cells, most of which were flat epithelial cells. Small round cells were a minority. The flat epithelial cells did not adhere to plastic and were eliminated from the culture after the first medium change on day 5-7. However, round cells did adhere to plastic and formed small colonies differing in size and morphology (Fig. 1). Most colonies consisted of fibroblastic cells; however some consisted of epithelioid cells. After the first medium change, the fibroblastic cell colonies started to proliferate actively, while the colonies with epithelioid cells did not change noticeably. Four to six days after the medium change, when the cells formed a subconfluent layer, the culture was replated; this did not lead to the formation of colonies. Cells distributed evenly on the bottom of the well and grew to confluence in 2-3 days. After passaging, the culture comprising only fibroblastic and epithelioid cells were no longer observed.

Three groups of AF cells may be distinguished according to their morphology: epithelioid, amniotic fluid-specific, and fibroblastic cells [13; 20]. The first group and the second one appear at the beginning of cultivation, and the third one appears later. The epithelioid cells quickly disappear, while the other two types persist in the culture. Many authors believe that the fibroblastic cells are stem cells and, taking into account the expression of mesenchymal markers and the spectrum of possible differentiations, place them into the MSCs category. However, other cells besides the fibroblastic cells can remain in the culture. These amniotic fluid-specific cells produce estrogen, progesterone, and chorionic gonadotropin. It seems that these cells originate from the trophoblast and the amniotic membrane. Thus, the question of amniotic stem cells origin is still unresolved [13; 20].

It is worth noting that we analyzed fast-adherent cells, as well as cells with delayed adhesion, because the first medium change was performed only 5-7 days after inoculation. Other researchers report slowly adhering cells (Tsai et al., 2004) and a mixed population, which was later selected for c-kit expression [7; 6; 12].

Some authors suggest that just c-kit positive cells in AF are pluripotent. Nevertheless, slowly adhering cells are also characterized by a wide range of markers and can differentiate into different types of cells.

It has been demonstrated that stem cells isolated from AF can undergo 350 population doublings maintaining their undifferentiated state, high proliferative potential, clonogenicity, and telomere length [17; 7; 12]. The maximum number of passages can reach 42 [22]. We have obtained three cultures from three donors. Currently, they have been cultivated to 20, 18, and 15 passages. According to a karyological analysis performed in a genetic laboratory, the fetuses did not exhibit any karyotype abnormality.

### Phenotype Characterization According to Molecular Marker Expression

**Fig. 2.**
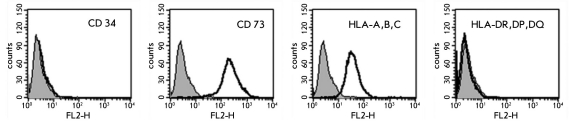
The results of flow cytometry analyses of CD34, CD73, HLA-A,B,C and HLA-DR,DP,DQ expression

Flow cytometry shows that cells from AF express the mesenchymal markers CD90, CD73, CD105, CD13, CD29, CD44, CD146, CD54, and CD71 (weak expression) and do not express CD106, CD34, or CD45 (Table 2, Fig. 2). These data suggest that fetal MSCs are present in our cultures. Judging by flow cytometry, the absence of CD34 and CD45 expression shows that there are no hemopoietic stem/progenitor cells in the culture. These results are in accordance with other authors, who report that hemopoietic stem cells are numerous in AF at the beginning of pregnancy (7-12 weeks). It seems that these cells get there through the thin wall of the yolk sac, where hemopoiesis is active at that time (Torricelli et al., 1993). At the usual time of amniocentesis (16-20 weeks), these cells are no longer present in AF [17; 6; 12; 16]. Keratin 19 was one of the epithelial markers discovered by flow cytometry and immunohistochemistry. Of the antigens of the major histocompatibility complex, (MHC), HLA-A,B,C were present and HLA-DR,DP,DQ were not, which is in accordance with the expression profiles of MSCs and AF cells [10; 20; 16; 26]).

**Table 2:** Flow cytometry analysis of marker expression in AF cells

Marker	Flow cytometrya *
CD 90 (Thy-1)	83% - 58% - 70%
CD 73 (SH3,SH4)	99% - 99% - 96%
CD 105	85% - 89% - n/a
CD 13	99% - 98% - 87%
CD 29 (integrin ≤1)	99% - 99% - 99%
CD 44	99% - 99% - 98%
CD 106 (VCAM-1)	- - -
CD 54 (ICAM-1)	43% - 60% - n/a
CD 146	99% - 98% - 90%
CD 71	36% - 32% - n/a
CD 34	- - -
CD 45	- - -
Keratin 19	92% - 70% - 88%
HLA-A,B,C	95% - 87% - 65%
HLA- DR,DP,DQ	- - -
This column quotes the percentage of cells positive for this marker in the three cultures, respectively; (-) no expression; and (n/a) was not measured.	

**Fig. 3.**
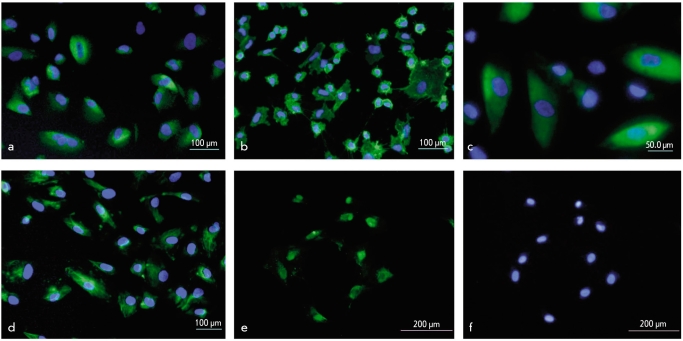
Immunohistochemical analyses of mesenchymal, neural and epithelial markers expression in cultured AF cells. Stained with antibodies against (a) CD105; (b) CD49d; (c) β_3_-tubulin; (d) keratin 19; (e) p63; (f) the same field, nuclei stained with DAPI. (a, b, c, d) Merge, nuclei stained with DAPI

**Fig. 4.**
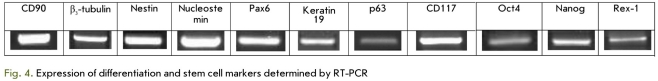
Expression of differentiation and stem cell markers determined by RT-PCR

Immunohistochemical analysis shows that AF cells express not only mesenchymal markers such as CD105, STR O-1, and CD49d, but also neural (neuronal cytoskeleton marker ≤3-tubulin, mature neuron neurofilament NF, and transcription factor Pax6) and epithelial markers (keratin 19, transcription factor p63) (Table 3, Fig. 3). Keratin 14, which is a marker of cornified epidermis, was not found in AF cells. Notably, more than 70% of the cells express keratin 19, and 80% also express the mesenchymal CD105 marker, and more than 95% express CD73. This suggests that a major portion of the cell population can express both mesenchymal and epithelial markers simultaneously. These data must be proven by alternative methods. It was demonstrated earlier that AF cell clones can express neural and mesenchymal markers simultaneously [21]. On the other hand, expression of neural markers in MSCs is also found [5; 23; 2]. However, keratin 19 expression is not character stic of mesenchymal cells. Thus, our data do not support the opinion that AF cells are fetal MSCs. RT-PCR data confirm that AF cells express the markers of mesenchymal (CD90), neural (≤3-tubulin, Nestin, Nucleostemin, Pax6), and epithelial (keratin 19, p63) lineages (Fig. 4). Also, RT-PCR detects the surface marker c-kit, homeobox gene Pitx2, and several ESCs markers (Oct4, Nanog, and Rex-1). These results and previous data [17; 21; 6] suggest a more primitive status of amniotic stem cells when compared to adult stem cells, as well as their wide differentiation potential. However, pluripotency markers such as Sox2 and Stella were not detectably expressed in AF cells. These data probably suggest that the potential of the studied cells is less than that of ESCs, and this might be the reason why AF cells do not form teratomas after in vivo transplantation.

**Table 3:** Immunohistochemistry analysis of marker expression in AF cells

Marker	Immunohistochemistry
CD 49d (integrin ≤4)	+
CD 105	+
STRO-1	+
CD 34	-
Pax6	+
NF	+
≤3-tubulin	+
Keratin 19	+
Keratin 14	-
p63	+
(+) marker expression detected; (-) marker expression not detected.	

### Transplantation of Cells into Immunodeficient Animals

Teratomas were not observed even 11 weeks after transplantation of cultured human AF cells into immunodeficient mice. Under the same conditions, human ESCs caused teratoma formation after 3-4 weeks. These results are in agreement with data, which show that the in vivo transplantation of a large number (up to 8 ° x 106) of AF cells does not cause teratoma formation [6; 19]. Furthermore, it was shown that even seven months after the intravenous injection of these cells, experimental animals had no detectable tumors [4].

Animals in the experimental group had no detectable organ abnormalities upon histological examination. Thus, we have obtained cell cultures from three AF donors. These cells are characterized by a wide spectrum of marker expression, including mesenchymal, neural, epithelial, and pluripotency markers.

Taking into account their morphology, cell culture behavior, and expression of multiple mesenchymal cell markers, one would assume that AF is a source of MSCs which are currently being discovered in many tissues [3; 9; 15]. There is data showing that MSCs can also express neural markers. On the other hand, the data obtained on the expression of epithelial markers suggests that the culture may either be heterogeneous or that AF cells may have a special differentiation status, which seems to be confirmed by the expression of pluripotency markers. We plan to investigate these possibilities in the future.

Current data on marker expression, differentiation potential of AF cells, and the absence of teratoma formation after in vivo transplantation leads us to the opinion that these cells are promising in the field of cell therapy. We suggest that AF cells can be useful for restoring spinal cord damage, treating diabetes, Alzheimer's disease, heart attack damage, etc. [8]. These cells can be used not only for autografts, but also for allografts, because the cells are only mildly immunogenic and donor-recipient pairs can be matched. Also, as opposed to other extraembryonic tissues (placenta, amniotic membrane, and umbilical blood) which are available only postnatally, AF cells can be obtained at 16-20 weeks of pregnancy. This suggests the possibility of treating developmental defects in utero or immediately after birth [11; 24]. 

## Acknowledgements

This work was supported by Grant from Russian Foundation for Basic Research (project 09-04-12132-ofi-m). 
